# Survey of *Dirofilaria immitis* antigen and antibodies to *Leishmania infantum* and *Toxoplasma gondii* in cats from Madeira Island, Portugal

**DOI:** 10.1186/s13071-020-3988-4

**Published:** 2020-04-21

**Authors:** Michelle Neves, Ana Patrícia Lopes, Carolina Martins, Raquel Fino, Cláudia Paixão, Liliana Damil, Clara Lima, Ana Margarida Alho, Henk D. F. H. Schallig, Jitender P. Dubey, Luís Cardoso

**Affiliations:** 1 University of Trás-os-Montes e Alto Douro (UTAD), Vila Real, Portugal; 2 Department of Veterinary Sciences, and Animal and Veterinary Research Centre (CECAV), UTAD, Vila Real, Portugal; 3 Sociedade Protetora dos Animais Domésticos (SPAD), Funchal, Portugal; 4 Department of Biological Sciences, Faculty of Pharmacy, Universidade do Porto, Oporto, Portugal; 5 CIISA, Faculty of Veterinary Medicine, University of Lisbon, Lisboa, Portugal; 6 Department of Medical Microbiology, Experimental Parasitology Section, Amsterdam University Medical Centres, Academic Medical Centre at the University of Amsterdam, Amsterdam, The Netherlands; 7 Animal Parasitic Diseases Laboratory, Beltsville Agricultural Research Center, Agricultural Research Service, US Department of Agriculture, Beltsville, MD United States of America

**Keywords:** Agglutination tests, Enzyme-linked immunosorbent assay, Epidemiology, Europe, Feline, Serology, Vector-borne pathogens, Zoonotic

## Abstract

**Background:**

*Dirofilaria immitis*, *Leishmania infantum* and *Toxoplasma gondii* are zoonotic parasites which can affect domestic cats. Considering the lack of published data from the local feline population, this study aimed to assess infection with or exposure to these pathogens in cats from Madeira Island, Portugal.

**Methods:**

One hundred and forty-one domestic cats (77 males and 64 females; median age: 2 years) were sampled at a veterinary medical centre in Funchal, from September 2018 to January 2019. Serum samples were tested for *D. immitis* antigen, with an enzyme-linked immunosorbent assay kit, and for antibodies to *Leishmania* spp. or to *T. gondii*, with the direct agglutination test and the modified agglutination test, respectively.

**Results:**

Five cats (3.5%; 95% confidence interval, CI: 1.2–8.1) were positive to *D. immitis*; no cats were seropositive to *Leishmania* spp. (0%; 95% CI: 0–2.6%); and 43 cats (30.5%; 95% CI: 23.0–38.8%) were seropositive to *T. gondii*. Prevalence of the *D. immitis* antigen was significantly different between cats that received ectoparasiticides and those which did not (0 *vs* 12.2%; *P* = 0.009). Prevalence of antibodies to *T. gondii* was significantly different between juvenile and adult cats (12.8 *vs* 38.0%; *P* = 0.007). There were two cats concurrently positive to *D. immitis* and *T. gondii*, but no statistical association between these two dependent variables was found (*P* = 0.641).

**Conclusions:**

To our knowledge, this is the first report of the presence of parasites *D. immitis* and *T. gondii* in the feline population of Madeira Island. Knowledge on the epidemiological situation of these and other zoonotic pathogens should raise awareness, both at the veterinary medical and public health levels, and contribute to promoting prevention and control. 
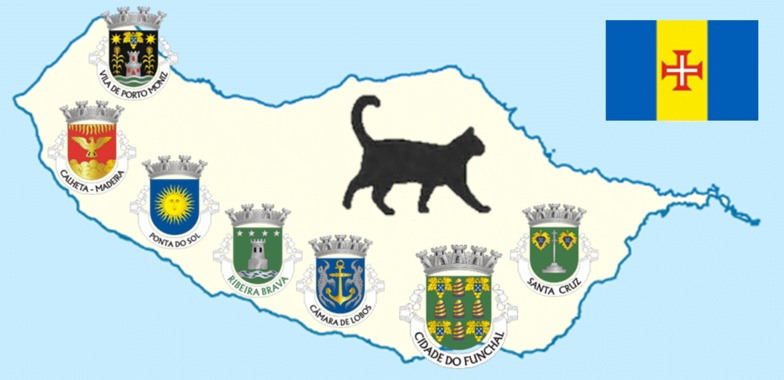

## Background

The nematode *Dirofilaria immitis* and protozoans *Leishmania infantum* and *Toxoplasma gondii* are zoonotic parasites which can affect domestic cats [1]. *Dirofilaria immitis* has mosquitoes as vectors, primarily from genera *Culex*, *Aedes* or *Anopheles*, and may cause cardiopulmonary dirofilariasis or heartworm disease, a potentially fatal illness in cats [2]. Illness usually develops as the so-called heartworm-associated respiratory disease (HARD) syndrome, then resulting in pulmonary inflammation and thromboembolism, often leading to fatal acute lung injury [3]. Clinical signs associated with feline cardiopulmonary dirofilariasis include tachypnea, coughing and increased respiratory effort, anorexia, vomiting, diarrhoea and weight loss, and occasional neurologic manifestations [4, 5]. Ascites, hydrothorax, chylothorax, pneumothorax, ataxia, seizures, and syncope have also been reported, but are not common [3]. Additionally, many cats have subclinical infections, i.e. without any detectable clinical signs, even knowing that only a small number of heartworms can compromise survival of their feline hosts [6, 7]. In humans, *D. immitis* might cause single or, more rarely, multiple pulmonary benign granulomas in peripheral areas occasionally misdiagnosed as malignant lesions [2, 8].

*Leishmania infantum* is transmitted mainly by phlebotomine sand flies among dogs, the primary reservoir of the protozoan parasite, but cats, humans and several other animals are also vertebrate hosts [9, 10]. Feline leishmaniosis caused by *L. infantum* predominantly manifests by distinct cutaneous lesions, although visceral and ocular involvement have also been reported [11, 12]. In Europe, human leishmaniosis is mainly observed in adults immunocompromised by HIV or immunosuppressants and in children [13, 14].

*Toxoplasma gondii* can infect almost all homeothermic animals as intermediate hosts, but domestic cats and other felids are the only definitive hosts of the parasite [15, 16]. After primary infection, infected cats may excrete millions of oocysts in their faeces into the environment, thus playing an important role in the spread of *T. gondii* [17]. Infections in domestic and wild cats are generally subclinical, but severe disease may occur, with interstitial pneumonia identified as a potential cause of mortality. Clinical findings may include ocular, gastrointestinal, hepatic, pancreatic, pulmonary and neuromuscular manifestations [18–20]. In immunocompromised adult humans and congenitally infected children, toxoplasmosis may be the cause of high morbidity and mortality, but infection in immunocompetent people is mainly subclinical [21, 22].

*Dirofilaria immitis*, *L. infantum* and *T. gondii* are pathogens endemic in animal and human populations from many parts of the world, including southern Europe [1]. However, no published information is available from the feline population on the Madeira archipelago, the southernmost territorial part of Portugal. Considering this lack of epidemiological data, the present report aimed at assessing the prevalence of *D. immitis* antigen and also those of antibodies to *L. infantum* and *T. gondii* in cats from Madeira Island.

## Methods

### Geographical area of the study

This study was conducted on Madeira Island, the main island of Madeira archipelago (a Portuguese autonomous region), on the African plate in the Atlantic Ocean, southwest of mainland Portugal. The island has an area of 741 km^2^ (53.9 and 23 km maximum length and width, respectively) and around 262,500 inhabitants, 130,000 of which live in the capital city, Funchal, the main urban center and port, on the south coast. In addition to Funchal, there are nine other municipalites on Madeira Island: Calheta, Câmara de Lobos, Machico, Ponta do Sol, Porto Moniz, Ribeira Brava, Santa Cruz, Santana and São Vicente. In general, Madeira Island has a temperate Mediterranean climate on the north coast and a subtropical dry climate on the south coast, with the economy being largely tourism-oriented. The monthly average air temperature is higher during summer (22.2 °C in August in Funchal) and lower in winter (15.9 °C in February in Funchal). Annual precipitation ranges from 553 mm in Funchal (58 m above sea level) to over 2000 mm in the north-facing slopes, increasing with altitude [23].

### Animals and samples

A total of 141 domestic cats were sampled at the veterinary medical centre of Sociedade Protetora dos Animais Domésticos (SPAD; Society for the Protection of Domestic Animals), in Funchal, from September 2018 to January 2019. The cats were brought in mainly for routine observation, clinical consultation, medical treatment or neutering surgery, by their owners, guardians or legal keepers, who signed an informed consent for inclusion of the animals in the study. Data available on the municipality of origin, age, sex, breed, fur length, housing, clinical status (regarding manifestations compatible with cardiopulmonary dirofilariasis, leishmaniosis and toxoplasmosis) and use of ectoparasiticides and macrocyclic lactones were registered for each cat (Table 1). Clinical manifestations looked for were anaemia, anorexia, ascites, ataxia, chylothorax, coughing, cutaneous lesions, diarrhoea, dyspnoea, fever, hydrothorax, increased respiratory effort, jaundice, pale mucous membranes, pneumothorax, seizures, and syncope, tachypnea, vomit, and weight loss. Minimum and maximum ages were 5 months and 21 years, respectively (median: 2 years; interquartile range: 0.7–6.0 years). All the cats had been born on Madeira Island and had no travelling history to mainland Portugal or any other destination. Blood samples (1 ml) were collected by jugular or cephalic venipuncture. Serum was separated by centrifugation and stored at − 20 °C until use.

**Table 1:** Prevalence of *Dirofilaria immitis* antigen and antibodies to *Toxoplasma gondii* in cats from Madeira Island, Portugal, according to the categories of several independent variables

Variable/category	No. (%) of cats tested	Percentage (*n*) of *D. immitis*-positive	Percentage (*n*) of *T. gondii*-positive
Municipality	141 (100)	*P* = 0.654^a^	*P* = 0.459^b^
Funchal	90 (63.8)	4.4 (4)	27.8 (25)
Other^j^	51 (36.2)	2.0 (1)	35.3 (18)
Age group	139 (98.6)	*P* = 0.322^a^	*P* = 0.007^c^
Juvenile [5–11 months]	39 (27.7)	0 (0)	12.8 (5)
Adult [1–21 years]	100 (70.9)	5.0 (5)	38.0 (38)
Sex	141 (100)	*P* = 1.0^a^	*P* = 0.067^d^
Female	64 (45.4)	3.1 (2)	39.1 (25)
Male	77 (54.6)	3.9 (3)	23.4 (18)
Breed	141 (100)	*P* = 1.0^a^	*P* = 0.247^a^
Mixed	133 (94.3)	3.8 (5)	29.3 (39)
Defined^k^	8 (5.7)	0 (0)	50.0 (4)
Fur length	138 (97.9)	*P* = 1.0^a^	*P* = 0.218^e^
Short	101 (71.6)	3.0 (3)	27.7 (28)
Medium	37 (26.2)	2.7 (1)	40.5 (15)
Housing	138 (97.9)	*P* = 1.0^a^	*P* = 0.081^f^
Indoors	34 (24.1)	2.9 (1)	17.6 (6)
Outdoors or mixed	104 (73.8)	3.8 (4)	35.6 (37)
Clinical status^l^	141 (100)	*P* = 0.134^a^	*P* = 0.705^g^
Non-suspect	122 (86.5)	2.5 (3)	29.5 (36)
Suspect	19 (13.5)	10.5 (2)	36.8 (7)
Ectoparasiticides^m^	103 (73.0)	*P* = 0.009^a^	*P* = 0.147^h^
Yes	62 (44.0)	0 (0)	25.8 (16)
No	41 (29.1)	12.2 (5)	41.5 (17)
Macrocyclic lactones^n^	107 (75.9)	*P* = 0.076^a^	*P* = 0.066^i^
Yes	44 (31.2)	0 (0)	18.2 (8)
No	63 (44.7)	7.9 (5)	36.5 (23)
Total	141 (100)	3.5 (5)	30.5 (43)

^a^Fisher’s exact test

^b^*χ*^2^ = 0.549, *df* = 1

^c^*χ*^2^ = 7.189, *df* = 1

^d^*χ*^2^ = 3.351, *df* = 1

^e^*χ*^2^ = 1.520, *df* = 1

^f^*χ*^2^ = 3.050, *df* = 1

^g^*χ*^2^ = 0.143, *df* = 1

^h^*χ*^2^ = 2.106, *df* = 1

^i^*χ*^2^ = 3.384, *df* = 1

^j^Calheta (*n* = 3; 2.1%), Câmara de Lobos (*n* = 19; 13.5%), Ponta do Sol (*n* = 4; 2.8%), Porto Moniz (*n* = 1; 0.7%), Ribeira Brava (*n* = 4; 2.8%) and Santa Cruz (*n* = 20; 14.2%)

^k^Persian (*n* = 1; 0.7%) and Siamese (*n* = 7; 5.0%)

^l^Clinical manifestations compatible with cardiopulmonary dirofilariosis and/or toxoplasmosis, comprising: anaemia, anorexia, diarrhoea, dyspnoea, fever, jaundice, pale mucous membranes and vomit

^m^Dinotefuran and pyriproxifen, fipronil and (S)-methoprene, fluralaner, lotilaner or spinosad

^n^Eprinomectin or milbemycin oxime

### Detection of *D. immitis* antigen

Serum samples were tested for *D. immitis* antigen with a commercial enzyme-linked immunosorbent assay kit according to the manufacturer’s instructions (PetChek Canine Heartworm Antigen Test®, IDEXX Laboratories, Westbrook, Maine, USA). This test detects antigen from live adult female heartworms or dying male (*n* ≥ 5) and female heartworms [3, 24]. Following the manufacturer’s instructions, a sample was considered qualitatively positive if it had more intense colour (blue) than the negative control; and all positive samples were retested for confirmation.

### Detection of antibodies to *L. infantum*

The direct agglutination test (DAT) for titration of IgG antibodies specific to *Leishmania* spp. used a standard freeze-dried antigen at a concentration of 5 × 10^7^ promastigotes per milliliter (Amsterdam University Medical Centres, Academic Medical Centre at the University of Amsterdam, Department of Medical Microbiology, Section Experimental Parasitology, Amsterdam, The Netherlands), following a predefined protocol [25]. Feline sera were diluted two-fold from 1:25 to 1:3200 in saline solution (0.9% NaCl) containing 0.1 M β-mercapto-ethanol, with a cut-off titre of 100 chosen for seropositivity [26]. Results obtained with DAT are expressed as an antibody titre, i.e. the reciprocal of the highest dilution at which agglutination (large diffuse blue mats) is still clearly visible after 18 h incubation at room temperature [25].

### Detection of antibodies to *T. gondii*

Sera were also tested for IgG antibodies to *T. gondii* at two-fold dilutions from 1:20 to 1:640 with a modified agglutination test (MAT) commercial kit (Toxo-Screen DA®, bioMérieux, Lyon, France) according to the manufacturer’s instructions. Positive and negative control samples, supplied with the kit, were included in each plate. Results obtained with the MAT were expressed as an antibody titre, i.e. the reciprocal of the highest dilution at which agglutination (at least one half of the well’s diameter) was still visible after 18 h incubation at room temperature. A cut-off titre of 20 (2 IU/ml in relation to a World Health Organization international reference serum) was chosen to maximize both sensitivity and specificity of the test [27, 28].

### Data analysis

Exact binomial 95% confidence intervals (CI) were established for prevalence values. The chi-square test and Fisher’s exact test (FET) were used to compare proportions of positivity among categories of the same independent variables and also total prevalence values of each agent. A *P* value ≤ 0.05 was considered as statistically significant. Analyses were performed with Stemstat, IBM SPSS Statistics 26^®^ software and WinEpi. Assuming a default 50% prevalence value and a 95% confidence level, a convenience sample of 141 cats corresponds to an absolute error of 8.25% [29, 30].

## Results

Five cats (3.5%; 95% CI: 1.2–8.1%) were found positive to *D. immitis* antigen (with all positive results confirmed by retesting); they were aged 2 years (*n* = 2), 5 years (*n* = 1), 6 years (*n* = 1) and 10 years-old (*n* = 1). Prevalence of *D. immitis* antigen was significantly different between cats that received ectoparasiticides and those which did not (0 *vs* 12.2%, respectively; *P* = 0.009; Table 1).

No cats were seropositive to *Leishmania* spp. (0%).

Forty-three cats (30.5%; 95% CI: 23.0–38.8%) were seropositive to *T. gondii*, with MAT titres of 20 in five, 40 in six, 80 in one and ≥ 640 in 31 cats. Thus, most seropositive cats (31/43; 72.1%) had a high titre (≥ 640). The 43 cats seropositive to *T. gondii* were aged from 6 months to 21 years-old. Prevalence of antibodies to *T. gondii* was significantly different between juvenile (5–11 months-old) and adult cats (1–21 years-old; 12.8 *vs* 38.0%, respectively; *P* = 0.007; Table 1).

Two cats were simultaneously positive to *D. immitis* and *T. gondii*, but no statistical association between these two dependent variables was found (FET: *P* = 0.641). Moreover, no statistically significant differences were found between the categories of the independent variables municipality, sex, breed, fur length, housing, clinical status and macrocyclic lactones, both for *D. immitis* and *T. gondii* (Table 1).

## Discussion

To the best of our knowledge, this is the first study describing the prevalence of infection with or exposure to vector-borne and zoonotic pathogens in the feline population from Madeira Island. The number of cats together with the incorporation of different animals may be considered a representative sample of Madeira Island, thus allowing an extrapolation of results to the regional feline population. No significant differences were found for the municipality, sex, breed, fur length, housing, clinical status and macrocyclic lactones (Table 1), suggesting that both *D. immitis* and *T. gondii* are uniformly distributed among the surveyed feline population of Madeira.

A few epidemiological studies have been performed in dogs from this region on *D. immitis* [8, 31] and *L. infantum* [31], but there were no published data regarding cats. Madeira is the region of Portugal with the highest prevalence of heartworm infection in dogs, in which cardiopulmonary dirofilariosis is the major endemic vector-borne disease [8, 31]. Although positivity could be expected among the feline population in a location where cardiopulmonary dirofilariasis is endemic in the canine population, this study documents *D. immitis* infection in cats on Madeira for the first time.

The 3.5% prevalence of *D. immitis* antigen in cats in the present study is significantly different from that of a recent report in dogs (40.0%; *χ*^2^  = 4.705, *df*  = 1, *P* < 0.0001) [31]. Cats are susceptible hosts, but they seem to be more resistant than dogs to infection with adult *D. immitis* [3]. According to another recent report [5], a theoretical prevalence of 9–18% of the infection prevalence in dogs is expected to be observed in cats. This assumption would suggest the prevalence of heartworm antigen in cats on Madeira to be 3.6–7.2%, which is in agreement with the value found in the regional feline population. Antigenaemia, mainly from adult female heartworms [32], is detectable in cats from around 5.5 to 8 months after natural infection [3]. Thus, six 5-month-old cats (data not shown) were not expected to be antigen-positive, a circumstance which increases the estimate of *D. immitis* antigen prevalence from 3.5% (5/141) to 3.7% (5/136).

Tests for detection of heartworm circulating antigens strongly depend on the number of mature adult females present in the host and cats usually have a much lower prevalence of adult infections than dogs [4]. A positive antigen detection test result is indicative of active adult infection, but a negative result does not rule out that the animal can be infected with only male, pre-adult or very old adult worms [5, 33]. Nevertheless, *D. immitis* antigen tests of the current generation identify most infections consisting of at least one mature female heartworm and are nearly 100% specific [3]. On the other hand, presence of antibodies to *D. immitis* may only indicate exposure to heartworm larvae rather than necessarily an active or persistent infection [4]. Tests for the detection of *D. immitis* antibodies detect infection earlier, but may also result in more false positives; *D. immitis* antigen tests detect infection later, but a positive result is generally confirmatory [3].

Transmission of heartworm is dependent upon several factors, including a suitable climate to support a viable mosquito population and the extrinsic incubation of *D. immitis* within those intermediate hosts [34]. The presence of *D. immitis* larvae in *Culex theileri* suggests that this mosquito species is actively involved as a natural vector of heartworm on Madeira [35]. An analysis of data from five meteorological stations in Portugal (years 2003–2013), revealed Madeira Island to be the region registering the highest number of days with suitable conditions for *D. immitis* transmission (average of 209.9 days per year) [36].

All the *D. immitis* antigen-positive cats (*n* = 5) did not receive ectoparasiticides. The products listed in Table 1 do not have a repellent or anti-feeding effect protecting against infective mosquito bites, but they are insecticides that contribute to reduce the total number or abundance of insects. Even if it could have been not regular, the application of ectoparasiticides was associated with a lower prevalence of infection in cats that received those products (*P* = 0.009; Table 1). In addition, none of the *D. immitis*-positive cats received macrocyclic lactones, but comparison with animals treated with them revealed a statistically non-significant difference, yet close to significance (*P* = 0.079; Table 1). Many cats may have received macrocyclic lactones not specifically for the prevention of *D. immitis*, but as a therapy against other endoparasites and even ectoparasites. Anyway, prevention of heartworm infection is attainable in cats by the routine administration of macrocyclic lactones, including eprinomectin and milbemycin oxime [37, 38]. Monthly chemoprophylaxis is a safe and effective option for cats living in geographical areas where *D. immitis* infection is considered endemic and exposure to infectious mosquitoes is likely [3].

The zero prevalence of antibodies to *Leishmania* spp. in cats from Madeira Island is in line with the value (0%) found in 10 apparently healthy dogs also from Madeira assessed, from October 2010 to April 2011, for *L. infantum* antibodies with a rapid test [31]. Some dogs living on Madeira were detected as seropositive to *Leishmania* spp. with the DAT, but those animals were born or had been in mainland Portugal (L. Cardoso, unpublished data), where zoonotic leishmaniosis caused by *L. infantum* is endemic [31, 39]. Specimens of *Phlebotomus sergenti* and *Sergentomyia minuta* have been found in Funchal, on Madeira Island [40], and on another island of the archipelago [41], respectively. The former species has been proven as vector of *Leishmania aethiopica* and *Leishmania tropica* [42], but its role as vector of *L. infantum* has never been confirmed [43]. The role of *S. minuta* in *Leishmania* spp. transmission among mammal hosts still needs to be elucidated [42]. Cases of canine leishmaniosis caused by *L. tropica* have been diagnosed in Morocco, in Africa, which is the continental country geographically closest to Madeira Island [44].

The 30.5% prevalence of antibodies to *T. gondii* found in the present study differs significantly (*χ*^2^ = 2.154, *df* = 1, *P* = 0.031) from the 20.5% found in domestic cats from the Lisbon metropolitan area, in mainland Portugal, using the same serological test [45]. This situation may due to the fact that different cut-offs have been used, i.e. a titre of 20 in the present study and a titre of 40 by the other authors [45]. Notwithstanding, the climacteric characteristics of Madeira might also mean more favourable conditions to the sporulation of *T. gondii* oocysts [16, 36]. On the other hand, a higher prevalence of *T. gondii* antibodies in adult (aged 1–21 years) in comparison with juvenile cats (5–11 months-old; Table 1) may be due to the fact that increasingly older cats have more chances to eat infected tissues or to contact with the surrounding environment potentially contaminated with oocysts [16]. Seropositive cats are likely to have already excreted *T. gondii* oocysts, whereby serological surveys for the detection of antibodies can help to determine the potential risk of infection in defined geographical areas [46, 47]. There are currently no data available on the seroprevalence of *T. gondii* from the canine population of Madeira.

## Conclusions

The present study provides evidence that cats on Madeira Island are at risk of becoming infected with *D. immitis* and *T. gondii*. On the other hand, no cats were found seropositive to *Leishmania* spp. Surveying these zoonotic parasites in the feline population of Madeira supplements their epidemiological mapping in cats from Portugal and Europe. In the scope of clinical practice, cardiopulmonary dirofilariasis and toxoplasmosis should be included in the differential diagnosis of feline patients with clinical signs compatible with both parasitoses.

## Data Availability

Data supporting the conclusions of this article are included within the article and its additional file. The datasets used and/or analysed during the present study are available from the corresponding author on reasonable request.

## Abbreviations

CI: confidence interval
DAT: direct agglutination test
FET: Fisher’s exact test
MAT: modified agglutination test
